# Antiviral effects of ferric ammonium citrate

**DOI:** 10.1038/s41421-018-0013-6

**Published:** 2018-03-27

**Authors:** Hongbin Wang, Zheng Li, Junling Niu, Yongfen Xu, Li Ma, Ailing Lu, Xun Wang, Zhikang Qian, Zhong Huang, Xia Jin, Qibin Leng, Jianhua Wang, Jin Zhong, Bing Sun, Guangxun Meng

**Affiliations:** 1 CAS Key Laboratory of Molecular Virology and Immunology, Institut Pasteur of Shanghai, Chinese Academy of Sciences, 200031 Shanghai, China; 2 Shanghai Blood Center, 200051 Shanghai, China

## Abstract

Iron is an essential nutrient for cell survival and is crucial for DNA replication, mitochondrial function and erythropoiesis. However, the immunological role of iron in viral infections has not been well defined. Here we found the iron salt ferric ammonium citrate (FAC) inhibited Influenza A virus, HIV virus, Zika virus, and Enterovirus 71 (EV71) infections. Of note, both iron ion and citrate ion were required for the antiviral capability of FAC, as other iron salts and citrates did not exhibit viral inhibition. Mechanistically, FAC inhibited viral infection through inducing viral fusion and blocking endosomal viral release. These were further evidenced by the fact that FAC induced liposome aggregation and intracellular vesicle fusion, which was associated with a unique iron-dependent cell death. Our results demonstrate a novel antiviral function of FAC and suggest a therapeutic potential for iron in the control of viral infections.

## Introduction

Iron element is required for multiple metabolic reactions in both eukaryotic and prokaryotic cells^1^. Meanwhile, iron also plays an important role in the immune system of vertebrates. During an infection process, the host utilizes various mechanisms to deprive iron for access by bacteria, which is termed nutritional immunity^2^. For example, lactoferrin secreted from neutrophils chelates iron in body fluids to inhibit the growth of the fungal pathogen *Aspergillus fumigates*^3^. Another secretary protein lipocalin 24p3 binds to *E. coli* siderophores and inhibits its absorption of iron^4^.

Ferric citrate is an iron salt formed by ferric ions and citrate. Citric acid, as a tricarboxylic acid cycle intermediate, is widely present in animals and plants. Human blood citric acid concentration is up to 100–120 μM^5^. Since citric acid is a natural iron chelating agent, the absorption and transport of iron by many bacteria and plants is accomplished in the form of ferric citrate^6,7^. Ferric citrate is also a form of non-transferrin-binding iron (NTBI) in plasma^8^. Although persistent high level of NTBI is associated with pathological conditions^8^, ferric citrate is safe as a drug and has been approved by the US Food and Drug Administration (FDA) for treatment of hyperphosphatemia^9,10^. A more dissolvable form of ferric citrate, ferric ammonium citrate (FAC) is used as food additive.

Influenza A virus (IAV) infection causes seasonal flu and pandemic flu worldwide and makes heavy socioeconomic burdens. There are more than 3 million severe cases and 0.25 million deaths caused by flu annually^11^. IAV is an enveloped negative-stranded RNA virus, which infects a wide variety of birds and mammals. According to the surface antigens hemagglutinin (HA) or neuraminidase (NA), IAVs are categorized into several subtypes, such as H1N1, H5N1, or H7N9^12^. The emergence of the highly pathogenic avian H5N1 influenza virus in 2004 and H7N9 IAV in 2013 raised concern of human influenza pandemic worldwide^13,14^. Flu vaccines can provide a good preventive effect, but the influenza virus mutations decrease the vaccine efficiency^15,16^. Although there are antiviral drugs on the market, due to the high variation rate of viral genome, high infectivity and high transmission speed of the virus, some influenza viruses have become resistant to antiviral drugs^12^. Thus it is still necessary to develop efficient and cheap new-generation drugs against IAV.

A clinic research on HIV-infected children with anemia reported that iron supplementation increased CD4^+^ T cell percentage, indicating a potential antiviral effect of iron^17^. In addition, it has been suggested that iron metabolism is associated with HCV and HIV infections^18^. Therefore, we set out to determine the immunological role of iron in viral infections in the current study, and found a significant inhibitory effect of FAC on multiple viruses including IAV, human immunodeficiency virus (HIV), Zika virus (ZIKV), and Enterovirus 71 (EV71).

## Results

### FAC inhibited IAV infection

**Fig. 1:**
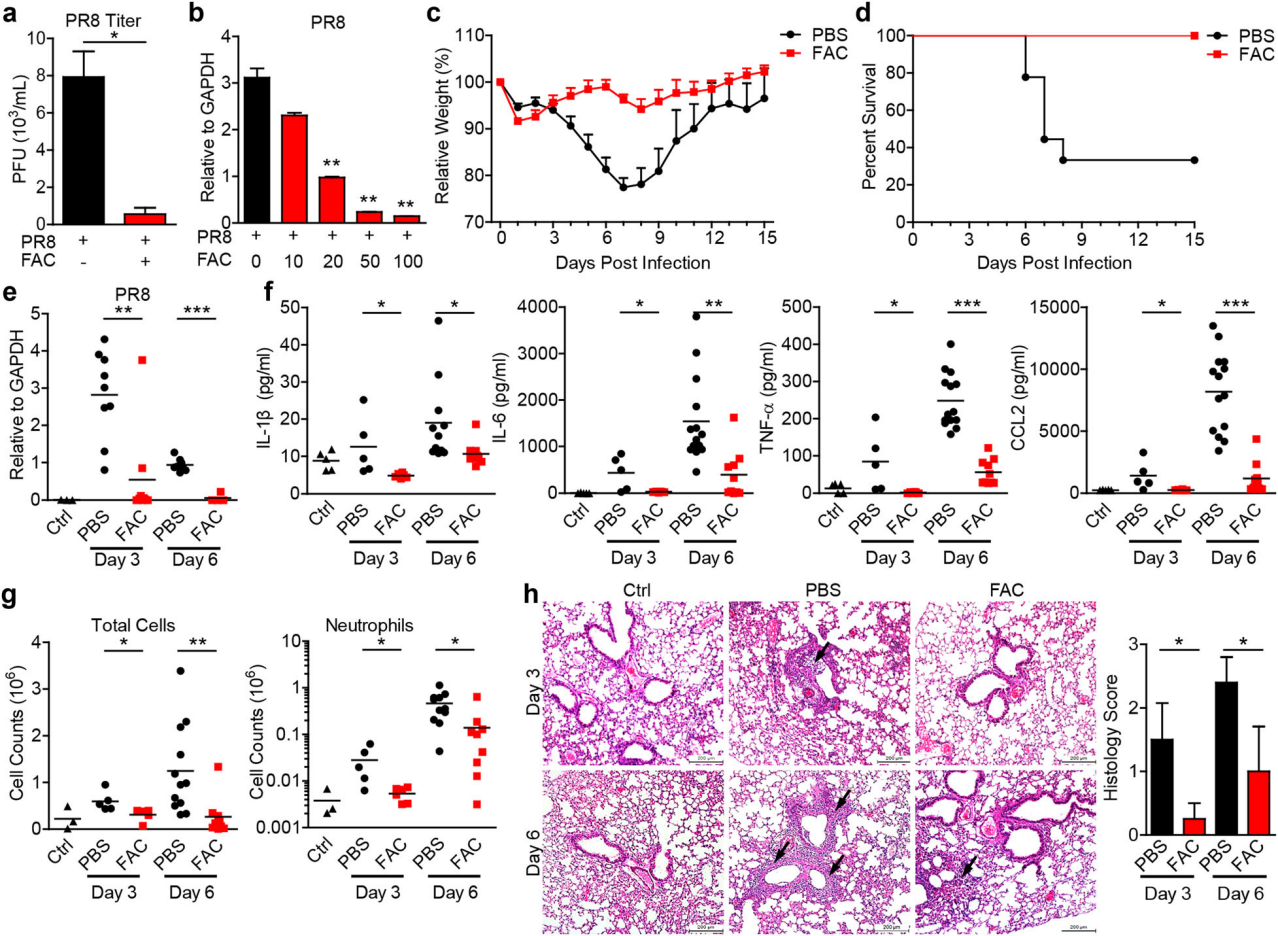
**FAC inhibited influenza A virus infection in vitro and in vivo. a** A549 cells were infected with PR8 (MOI = 0.1) ± (with or without) FAC (100 μM). 24 h later, supernatants were subject to viral titer assay. **b** A549 cells were infected with PR8 (MOI = 0.1), treated with increasing doses of FAC (μM). 12 h later, viral RNA loading was analyzed through real-time PCR. **c**,** d** 6–8-week old female C57BL/6 mice were intranasally infected with 1 × LD_50_ influenza A virus PR8 diluted in 25 μl PBS or PBS with FAC (15 mM) (each group, *n* = 9). Mice weight change (**c**) and survival rate (**d**) were recorded. Mice with relative weight lower than 75% were euthanatized and considered as death. *P* < 0.0001 in (**c**) (PBS versus FAC, 2-way ANOVA test). *P* = 0.0033 in (**d**) (PBS versus FAC, log-rank (Mantel-Cox) test). **e**–**h** Mice were infected as in (**c**) 25 μl PBS or PBS with FAC (15 mM) was intranasally delivered at day 2 post infection. Whole lung and bronchoalveolar lavage fluids (BALFs) of mice were collected at days 3 and 6. The viral RNA loading in lung was analyzed by real-time PCR (**e**). IL-1β, IL-6, TNF-α, and CCL2 in the whole lung and BALF were analyzed via ELISA (**f**). Infiltrated total cells and neutrophils (Ly-6G^+^) in BALFs were analyzed through flow cytometry (**g**). Lung tissue inflammation was visualized via H&E staining (left) (**h**), arrows indicate tissue damage. Ctrl, mice without infection or treatment; Scale bars, 200 μm. Histological scores (right) were made by estimating lung inflammation levels by a pathologist blinded to the study (0 = absent; 1 = light; 2 = moderate; 3 = severe). Data are representative of at least two independent experiments in (**a**, **b**). Error bars represent SEM in (**c**) or SD in other panels

We firstly found that during influenza A virus PR8 infection of human lung epithelial cell line A549 cells, ferric ammonium citrate (FAC) (100 μM) significantly blocked PR8 replication and virus-induced expression of innate immune factors (Fig. 1a and Fig. S1A). Dose dependence assay showed that FAC concentration negatively correlated with PR8 replication (Fig. 1b). To demonstrate whether FAC is toxic to the cells, we treated cells with increased concentrations of FAC and detected the release of the cell death indicator lactate dehydrogenase (LDH). Results of this experiment showed that even high concentration of FAC (10 mM) did not induce cell death (Fig. S1B). In human monocytic THP-1-derived macrophages, FAC also inhibited PR8 replication and virus-induced proinflammatory cytokine IL-1β secretion (Figs. S1C and S1D).

Next we determined the antiviral effect of ferric citrate in vivo. Influenza A virus and FAC (15 mM) were co-inoculated into airway of mice to test FAC’s antiviral effect. We found that FAC dramatically protected mice from influenza PR8 virus infection reflected by weight loss and survival rate (Fig. 1c, d). Additional analysis showed that FAC dampened viral RNA replication, as well as the expression of proinflammatory cytokines, IFN-β and chemokines in the lung of infected mice (Fig. 1e and Fig. S1E). In the bronchoalveolar lavage fluid (BALF), the levels of proinflammatory cytokines decreased considerably (Fig. 1f), and there were decreased levels of migrated total cells and neutrophils in FAC-treated mice (Fig. 1g). Moreover, H&E staining of lung tissue showed an alleviated lung inflammation in FAC-treated mice (Fig. 1h). Taken together, these results demonstrate that ferric ammonium citrate is able to control IAV infection in vitro and in vivo.

### FAC inhibited HIV, ZIKV, and EV71 infection

**Fig. 2:**
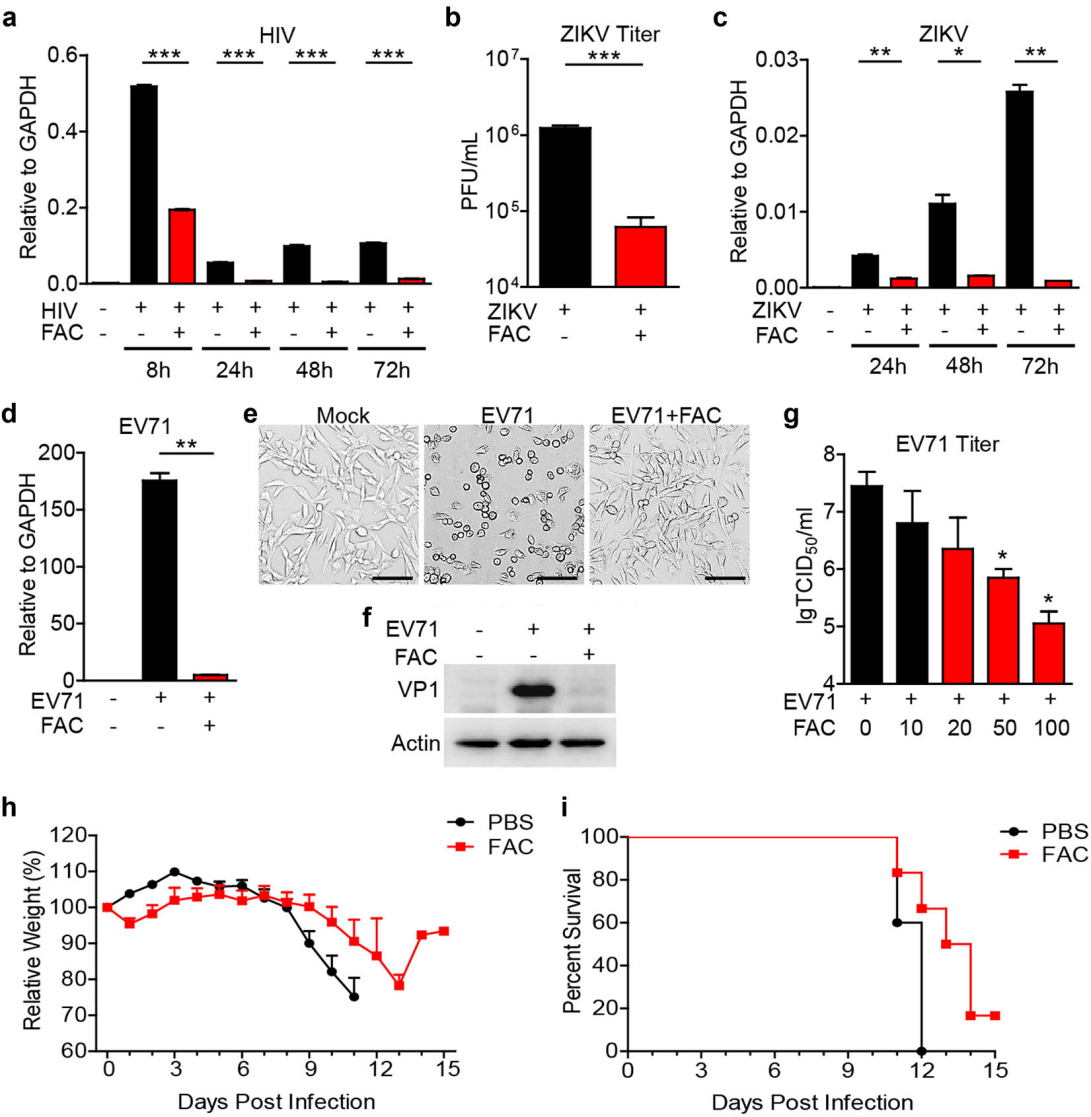
**FAC inhibited HIV, ZIKV and EV71 infections.** **a** Human PBMC-differentiated dendritic cells were infected with HIV-1 (Gag/p24 100 ng/ml) ± FAC (100 μM). 8, 24, 48, and 72 h later, HIV Gag RNA loading was analyzed through real-time PCR. **b** Vero cells were infected with ZIKV (MOI = 0.01) ± FAC (100 μM). Seventy two-hour later, viral titers in supernatants were analyzed. **c** U251 cells were infected with Zika virus (MOI = 0.1) ± FAC (100 μM). 24, 48 and 72 h later, viral RNA loading was analyzed through real-time PCR. **d**–**f** RD cells were infected with EV71 (MOI = 0.1) ± FAC (100 μM) for 8 h. Viral RNA loading was detected via real-time PCR (**d**). Cytopathy images were captured through bright field microscopy (**e**). Scale bars, 100 μm. Viral VP1 protein expression was detected via western blot (**f**). **g** RD cells were infected with EV71 (MOI = 0.1) and treated with increasing doses of FAC (μM). 1 h later, viruses were washed out and FAC was supplemented. 9 h later, viral titers in supernatant were analyzed through TCID50 method. **h**, **i** 6–8-week old female IFNRα/β^−/−^IFNγ^−/−^ mice were intraperitoneally infected with 40 PFU ZIKV diluted in 800 μl PBS or PBS with FAC (15 mM) (each group, *n* = 5). Mice weight change (**h**) and survival rate (**i**) were recorded. *P* < 0.0001 in (**h**) (PBS versus FAC, 2-way ANOVA test). *P* < 0.05 in (**i**) (PBS versus FAC, log-rank (Mantel-Cox) test). Data are representative of at least two independent experiments in (**a**–**g**). Error bars represent SEM in (**h**) or SD in other panels

Next, we tested FAC’s effect on other viruses. Human immunodeficiency virus (HIV) infection causes the acquired immunodeficiency syndrome (AIDS), a chronic disease carried by more than 35 million people worldwide^19^. HIV specifically infects immune cells such as CD4^+^ T cells, macrophages, and dendritic cells, leading to the progressive destruction of the immune system. As a result, pathogen infections may lead to the death of the patients. We found that FAC inhibited HIV-1 infection of human dendritic cells at various time points (Fig. 2a). Accordingly, virus-induced innate immune response was inhibited by FAC (Fig. S2A).

Zika virus (ZIKV) is a positive single-stranded RNA virus in the Flaviviridae family^20^. Mosquito-borne ZIKV infection of adults typically causes low fever, rash, joint pain, conjunctivitis, myalgia, headache, and weakness. Infection of pregnant women transmits the virus to the fetus, causing severe neonatal head malformations^21^. The recent epidemics of ZIKV infection in Central and South America have sparked the attention of society and the scientific community^22^. There is no specific treatment for the virus at present, thus it is urgent to develop specific anti-ZIKV drugs. We found FAC also inhibited Zika virus replication in various cell systems (Fig. 2b, c and Fig. S2B).

Enterovirus 71 (EV71) is a highly pathogenic non-enveloped single-stranded RNA virus from Picornaviridae family^23^. EV71 infection mainly causes hand, foot, and mouth disease (HFMD), as well as severe complications such as encephalitis and neurogenic pulmonary edema^24^. However, there is no effective antiviral treatment for severe EV71 infection cases yet^23^. Treatment of FAC dramatically dampened virus genome amplification (Fig. 2d), virus-induced cytopathy (Fig. 2e) and virus VP1 protein expression (Fig. 2f) in rhabdomyosarcoma (RD) cells. Dose dependence was observed for mature virion release and intracellular viral RNA loading (Fig. 2g and Fig. S2C). In THP-1 macrophages, EV71 replication activated the NLRP3 inflammasome leading to activation of Caspase-1, which cleaves pro-IL-1β to its mature form for secretion^25^. FAC treatment decreased EV71 replication and IL-1β secretion in such cells (Figs. S2D and S2E). Immunoblot assay further confirmed that FAC blocked EV71-induced secretion of mature Caspase-1 and IL-1β, as well as the oligomerization of the NLRP3 inflammasome adapter protein ASC (Fig. S2F). Finally, using mouse ZIKV infection model, we found that co-inoculation of FAC (15 mM) and ZIKV inhibited the weight loss (Fig. 2h) and increased the survival rate of experimental animals compared to the controls infected with ZIKV only (Fig. 2i). All these results indicate that ferric citrate has the ability to inhibit intracellular viral replication and therefore deplete virus-induced immune responses.

### Specific iron ion and citrate combinations inhibited viral infection

**Fig. 3:**
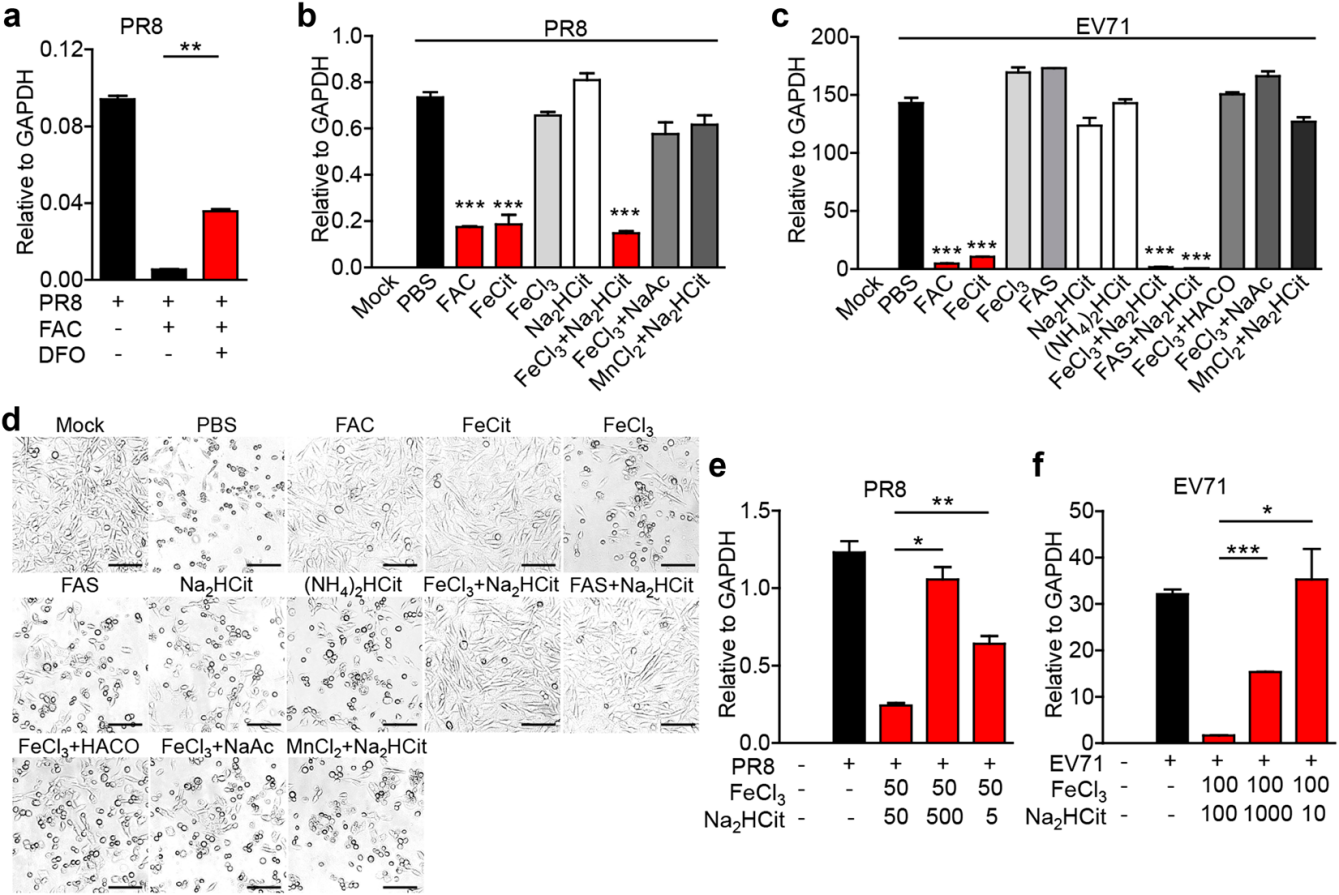
**Specific iron salt inhibited viral infection.** **a** PR8 (MOI = 0.1)-infected A549 cells were treated with FAC (100 μM) and deferoxamine (DFO, 100 μM). 10 h later, viral RNA was analyzed through real-time PCR. **b** A549 cells were infected with PR8 and treated with indicated compounds (50 μM) or compounds combinations (50 μM + 50 μM). 12 h later, viral RNA was detected via real-time PCR. **c**, **d** RD cells were infected with EV71 and treated with indicated compounds (100 μM) or compounds combinations (100 μM + 100 μM). 12 h later, viral RNA was detected via real-time PCR (**c**); cytopathy images were captured through bright field microscopy (**d**). Scale bars, 100 μm. **e** A549 cells were infected as in (**b**) and treated with indicated compounds combinations (μM). Viral RNA was detected via real-time PCR. **f** RD cells were infected with EV71 and treated with indicated compounds combinations (μM). Twelve-hour later, viral RNA was detected via real-time PCR. Data are representative of three independent experiments. Error bars represent SD

In solution, ferric citrate forms various complexes, thus is endowed with functions similar to small molecule drugs^26^. To understand the relative contribution of ferric cation and citrate anion to the inhibitory effect demonstrated above, we first treated cells with deferoxamine (DFO), an iron chelator, and found DFO rescued FAC-restricted PR8 replication (Fig. 3a), confirming the necessity of iron. Intriguingly, FAC and ferric citrate (FeCit) but not ferric chloride (FeCl_3_) or disodium citrate (Na_2_HCit) had the anti-PR8 effect (Fig. 3b), indicating that both ferric ion and citrate ion are indispensable. Indeed, FeCl_3_ combined with Na_2_HCit, but not FeCl_3_ combined with sodium acetate (NaAc), or manganese (II) chloride (MnCl_2_) combined with Na_2_HCit exhibited the anti-PR8 effect (Fig. 3b). In the EV71 infection system, we also found FAC, FeCit, FeCl_3_ combined with Na_2_HCit, and ferric ammonium sulfate (FAS) combined with Na_2_HCit, but not FeCl_3_, FAS, Na_2_HCit, ammonium citrate dibasic ((NH_4_)_2_HCit), FeCl_3_ combined with cis-aconitic acid (HACO), FeCl_3_ combined with NaAc, or MnCl_2_ combined with Na_2_HCit had anti-EV71 effect (Fig. 3c, d). The molar ratio of ferric ion to citrate ion can affect ferric citrate complex speciation^6^. We found that when ferric ion and citrate ion ratio was adjusted to 1:10 or 10:1, the antiviral effect decreased significantly (Fig. 3e, f). These results suggest that specific ferric citrate complexes were required for the antiviral activity.

### FAC-induced virus fusion

**Fig. 4:**
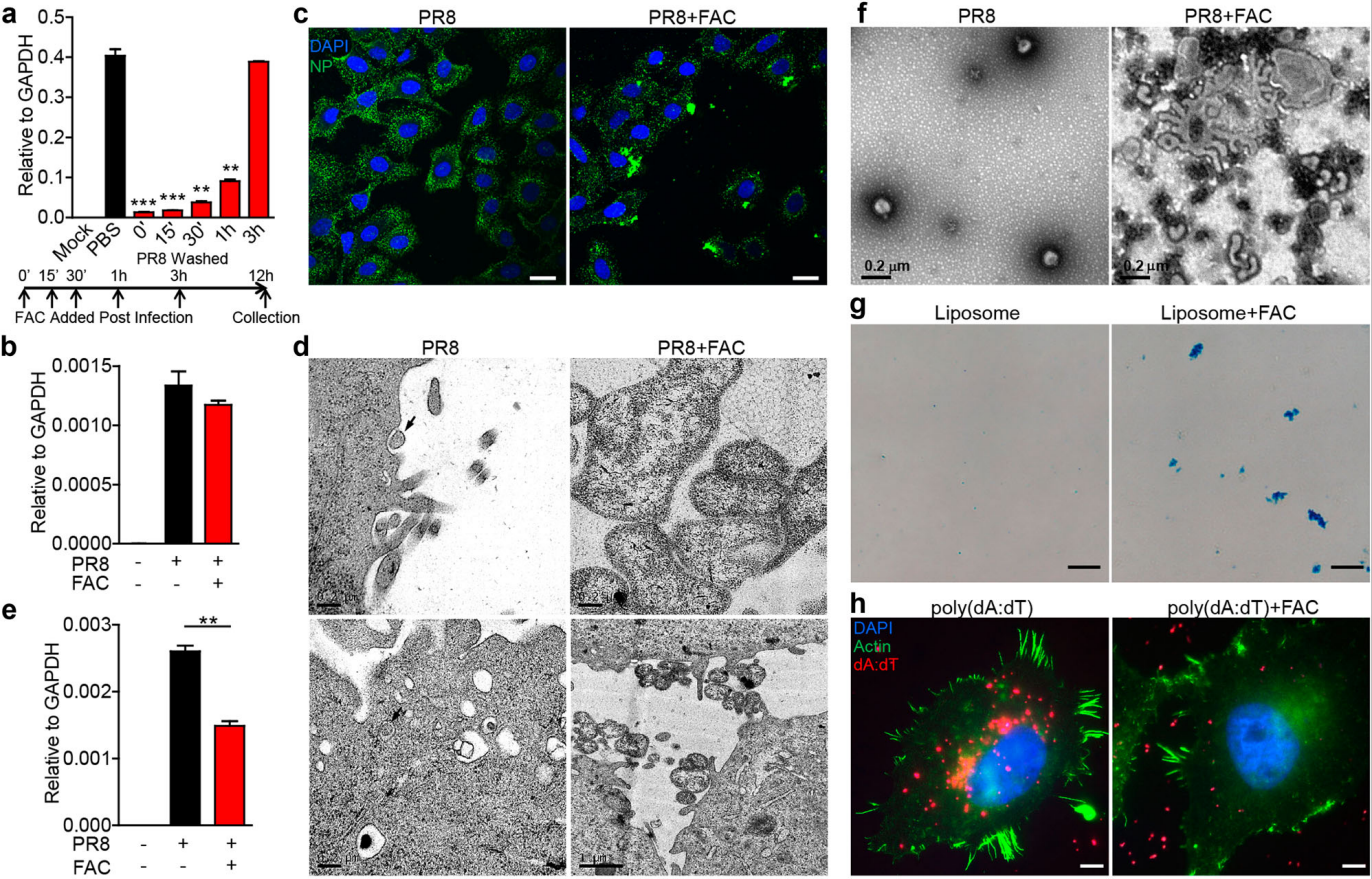
**FAC-induced fusion of influenza A virus or liposome.** **a** PR8-infected A549 cells were treated with FAC at indicated time points post infection. Viruses were washed out at 3 h post infection and cells were analyzed for viral RNA at 12 h post infection through real-time PCR. **b** A549 cells and PR8 were incubated at 4 °C ± FAC for 1 h and cells were washed and analyzed for viral RNA through real-time PCR. **c** PR8-infected A549 cells were treated with or without FAC for 1 h. Cells were analyzed under confocal microscopy of PR8 NP protein. Scale bars, 20 μm. **d** PR8-infected A549 cells were treated in the presence or absence of FAC for 1 h. Cells were analyzed via electron microscopy. Arrows indicate endocytosing and endocytosed viruses in the PR8 panels (left, upper and lower images, scale bars: 0.2 μm). In the PR8 + FAC panels, upper image shows fusion viruses (right, upper panel, scale bar: 0.2 μm); lower image shows fusion viruses on the cell surface (right, lower panel, scale bar: 1 μm). **e** A549 cells and PR8 virus were incubated at 37 °C ± FAC for 3 h. Cells were washed and analyzed for viral RNA through real-time PCR. **f** PR8 in solution with or without FAC was incubated for 1 h at room temperature, centrifuged, and analyzed by negative-stain electron microscopy. **g** Liposome ± FAC were incubated for 1 h at 37 °C, stained with DiD (50 μM), and analyzed through bright field microscopy. Scale bars, 20 μm. **h** Hela cells were transfected by liposome and poly(dA:dT)-rhodamine mix ± FAC for 6 h. Cells were analyzed via confocal microscopy. Scale bars, 10 μm. Data are representative of three independent experiments. Error bars represent SD

We next investigated the underlying mechanism for the antiviral effect of FAC and found that although FAC inhibited PR8 replication at early time points, it lost the inhibitory effect if added 3 h post infection (Fig. 4a), suggesting that FAC targeted early events during viral infection. Similar result was observed in EV71 infection (Fig. S3A). Virus attachment assays showed that FAC did not inhibit PR8 or Zika virus binding to host cells (Fig. 4b and Fig. S3B). We further investigated whether FAC could interfere with cellular endocytosis. Transferrin and Cholera Toxin Subunit B (CTB) enter cells through clathrin-dependent and caveola-dependent endocytosis, respectively^27,28^. FAC did not affect these two endocytosis routes (Fig. S3C). These results indicate that viral entry into endosome is not the target of FAC.

Notably, when the influenza virus was visualized under immunofluorescence microscopy, we found FAC-induced PR8 aggregation on cell surface (Fig. 4c and Fig. S3D). Further analysis with transmission electron microscopy showed that at 1 h post infection, PR8 viruses were either in the process of endocytosis or had been endocytosed into cells, whereas FAC treatment-induced fusion of virions on the plasma membrane, which made the virions difficult to be endocytosed by the cell (Fig. 4d). This explained the fact that FAC partially inhibited PR8 endocytosis at 3 h post infection (Fig. 4e). To understand whether there was a direct effect on virus, we co-incubated PR8 virus with FAC and negative-stain electron microscopy images showed that FAC directly induced viral fusion (Fig. 4f).

FAC might bind and connect viral membranes to induce fusion. To understand the specificity, we tested FAC’s role on liposome, and found FAC also induced liposome aggregation (Fig. 4g). Furthermore, liposome-mediated DNA or RNA transfection was blocked by FAC (Fig. 4h and Fig. S3E). As a result, expression of liposome-delivered plasmid was depleted accordingly (Fig. S3F). By contrast, electroporation-dependent gene transfection was not affected by FAC (Fig. S3G). These data demonstrate that FAC promotes membrane fusion.

### FAC inhibited endosomal release of viruses

**Fig. 5:**
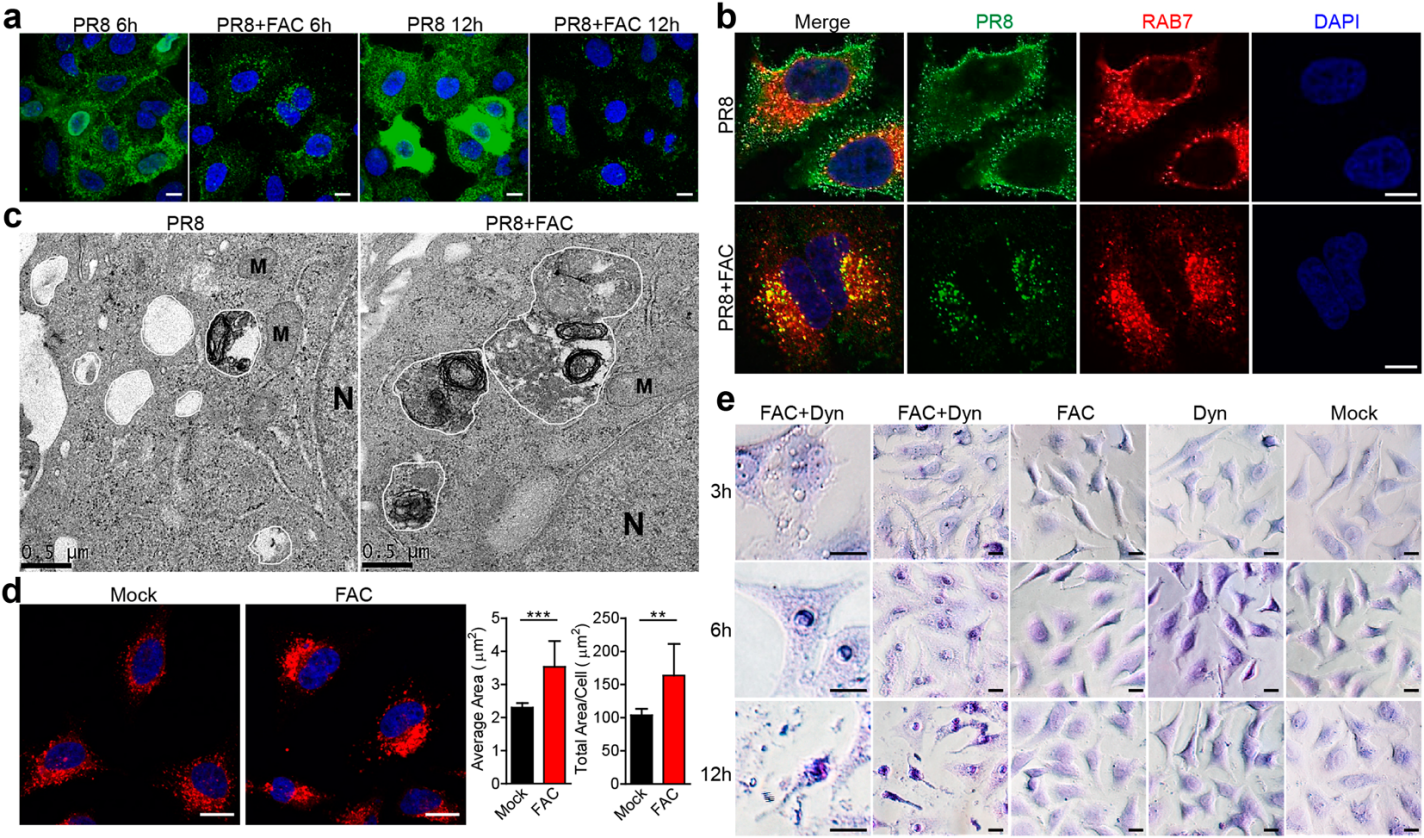
**FAC-induced intracellular vesicle fusion.** **a** PR8-infected A549 cells were treated in the presence or absence of FAC for 6 h or 12 h. Cells were analyzed under confocal microscopy for PR8 NP protein. Scale bars, 20 μm. **b** PR8-infected Hela cells were treated in the presence or absence of FAC for 6 h. Cells were analyzed under confocal microscopy for PR8 NP protein and RAB7. Scale bars, 10 μm. **c** A549 cells were infected with PR8 ± FAC for 6 h and analyzed via electron microscopy. Endosomes (irregular circles, white inside) and endolysosomes (irregular circles, dark inside) were marked by white lines. N nucleus. M mitochondrion. **d** Hela cells were treated with or without FAC for 6 h and analyzed through confocal microscopy for RAB7. Average RAB7-positive vesicle area and total RAB7 positive vesicle area per cell were statistically analyzed. Scale bars, 20 μm. **e** Hela cells were treated with Dynasore (Dyn) and/or FAC for indicated time. Cells were stained with 0.1% crystal violet and analyzed under bright field microscopy. Scale bars, 20 μm. Data are representative of three independent experiments. Error bars represent SD

Although FAC-induced viral fusion, considerable amount of viral particles still entered the cell. Confocal microscopy analysis showed that PR8’s entry into RAB5-expressing early endosomes was not affected by FAC during early infection (Fig. S4A). At 6 h post infection, endocytosed PR8 viruses entered cytosol, and viral nucleoprotein (NP) moved into nucleus to initiate replication; whereas in FAC-treated cells, PR8 viruses were kept in intracellular vesicles (Fig. 5a). At 12 h post infection, NP proteins were mass produced for virion assembly; whereas in FAC-treated cells, PR8 viruses were still trapped in vesicles without NP protein expression (Fig. 5a). Similar result was observed on EV71 (Fig. S4B). Further analysis showed that the trapped PR8 viruses co-localized with RAB7, a late endosome marker (Fig. 5b, Figs. S4C, S4D), indicating that in the presence of FAC, PR8 viruses were not able to escape from late endosome and translocate into cytosol. This was not due to a defect of PR8 hemagglutinin (HA) protein-induced membrane fusion as shown in Fig. S4E that FAC or PBS treatment did not result in any difference in HA-mediated membrane fusion. Transmission electron microscopy images showed that at 6 h post infection, FAC-treated cells exhibited large endolysosomal vesicles full of digested components (probably the PR8 remains); whereas in untreated cells, there were relatively small and empty endosomal or endolysosomal vesicles (Fig. 5c), indicating that viruses had escaped into the cytosol. This phenomenon was through FAC’s direct impact on host cells, because FAC alone induced enlarged RAB7-expressing endosomes in Hela and A549 cells (Fig. 5d and Fig. S4F), indicating that FAC is also able to enhance intracellular vesicle fusion.

Dynasore, a dynamin inhibitor, inhibits cellular endocytosis and induces stabilized pits on plasma membrane^29^. These stabilized pits may have endosome features. Of note, co-administration of Dynasore and FAC induced crater-like depressions on cell surface (Fig. 5e and Fig. S5A, upper panels), indicating that FAC-dependent vesicle fusion expanded Dynasore-induced pits into large craters accompanied with enhanced exocytosis. This was evidenced by the fact that cytosolic components were dramatically exocytosed after long-time treatment, leading to a flat and thin cellular morphology and a pyknotic nucleus (Fig. 5e and Fig. S5A, middle panels). This process was reversible when Dynasore and FAC were washed and replaced by normal medium (Fig. S5B). Moreover, continued treatment caused cell death (Fig. 5e and Fig. S5A, lower panels). Taken together, these results demonstrate that FAC affects intracellular vesicles to interfere with viral infection.

## Discussion

Our present study found that an iron salt ferric ammonium citrate (FAC) inhibited the infection of influenza A virus (IAV), human immunodeficiency virus (HIV), Zika virus (ZIKV), and Enterovirus 71 (EV71), suggesting that iron is involved not only in nutritional metabolism but also in antiviral immunity. Of note, both ferric ion and citrate ion are essential for antiviral effects. Mechanistically, FAC directly induces the fusion of viral membranes, or inhibits the escape of viruses from endosomes through inducing fusion of vesicles in host cells.

The detailed mechanism of FAC-induced viral membrane fusion still needs to be explored. Since FAC can also induce liposome aggregation, it seems that FAC can directly target lipid molecule instead of viral envelope proteins to induce the fusion. It is known that ferric ion is a phosphate binder^30,31^. Thus it is possible that FAC induces membrane fusion through a model that ferric ion binds phosphate head group of phospholipid molecules such as phosphatidylcholine and phosphatidylserine, while citric radical inserts into lipid bilayer to bridge membranes.

Likewise, the intracellular vesicle fusion of vesicles induced by FAC is possibly mediated by the same viral fusion mechanism. However, it is still possible that FAC interferes with intracellular proteins involved in vesicle traffic, fission or fusion. We have applied magnetic beads-based pull-down of ferric citrate binding proteins coupled with mass spectrometry, and identified a group of organic cyclic compound binding proteins (data not shown). Further analysis of these proteins may give us new insight into the mechanism.

Iron-related drug development has been focused on iron metabolism but neglected its direct impact on viruses^32^. Our current study delineates an antiviral role of the iron salt FAC on multiple viruses such as influenza A virus (IAV), human immunodeficiency virus (HIV), Zika virus (ZIKV), and Enterovirus 71 (EV71), which is stimulating for antiviral drug development.

Since FAC directly targets virus and inhibits early viral infection events, it is useful to be applied as viral-prevention agents; but may not be effective when applied after establishment of viral infection in the host. When we tested the time window for application of FAC in vivo for prevention (FAC 30 min, then PR8 infection) or therapeutic (PR8 infection, then FAC 30 min) purposes, FAC had no inhibitory effect on the virus (data not shown). Moreover, we also need to keep in mind that large amounts of iron chelating proteins such as transferrins are present in the circulation, which may interfere with systemic application of iron for therapeutic purpose. In addition, oral administration of high dose of ferric citrate is associated with gastrointestinal side effects such as constipation and diarrhea^33^. Thus, alternative administration route should be considered for iron application to human body, which may include but not limited to spray, liniment or suppository.

## Experimental procedures

### Compounds and chemicals

All iron related compounds were from Sigma. Dynasore (D7693, Sigma), DFO (D9533, Sigma). Butylated hydroxyanisole (BHA, B1253, Sigma), N-acetyl-l-cysteine (NAC, A7250 Sigma), diphenyleneiodonium (DPI, D2926 Sigma), Mito-TEMPO (SML0737, Sigma).

### Cells

All cell lines were obtained from ATCC and free of mycoplasma. A549 and THP-1 cells were grown in RPMI medium (SH30809, HyClone) supplemented with 10% (v/v) FBS (FSP500, ExCell Biology) and 1% (v/v) Penicillin-Streptomycin (15140122, Thermo Fisher Scientific). RD, U251, Vero, Hela, HEK293T, and L929 cells were grown in DMEM (SH30243.01, HyClone) supplemented with 10% FBS and 1% Penicillin-Streptomycin. Cells were incubated at 37 °C in a 5% CO_2_ incubator (Thermo Scientific). For THP-1 macrophage differentiation, cells were treated with 50 ng/ml Phorbol 12-myristate 13-acetate (PMA, P8139, Sigma) for 2 h and rested for 24 h to become adherent macrophages.

Preparation of human monocyte-derived dendritic cells (MoDCs) had been described previously^34^. Briefly, primary human peripheral blood mononuclear cells (PBMCs) were isolated from buffy coats of healthy donors using Ficoll-Paque PLUS (GE Healthcare Life Sciences). CD14^+^ monocytes were isolated from PBMCs by MACS using magnetic microbeads (Miltenyi Biotec) and cultured in RPMI 1640 (Life Technologies) supplemented with 10% FBS, 1% P/S, 50 ng/mL GM-CSF (R&D Systems), and 40 ng/mL IL-4 (R&D Systems) to differentiate for 6 days into immature MoDCs. The Ethics Committee of Institute Pasteur of Shanghai approved the HIV-1 infection experiment on human MoDCs.

### Viruses and cell infection

Influenza A virus (strain A/Puerto Rico/8/1934 H1N1, PR8) was described before^35^. For infection of A549 cells or THP-1 macrophages with PR8, the medium was serum free.

HIV-1 virus was described before^36^. For HIV-1 infection of MoDCs, viruses was added into MoDC culture medium at a concentration of Gag/p24 = 100 ng/ml. At various time points post infection, cells were lysed in TRIzol reagent (Invitrogen) to isolate RNA according to the manufacturer’s instructions.

Zika virus/SZ-WIV01/2016/China was obtained from Center for Emerging Infectious Diseases, Wuhan Institute of Virology, Chinese Academy of Sciences, Wuhan, China. Virus stocks were propagated in African green monkey kidney cell line Vero. The virus infection assay was performed in human glioma cell line U251MG and Vero cells at a multiplicity of infection (MOI) of 0.1. At various time points post infection, cells were lysed in TRIzol reagent to isolate RNA.

The EV71 FY573 isolate (subgenotype C4a, GenBank accession number: HM064456.1) was reported before^25^. Viral titer in the supernatant was determined through TCID_50_ assay.

Viral infection experiments followed the standard operating protocols approved by the Institutional Biosafety Committee and were performed in biosafety level 2 laboratory at Institut Pasteur of Shanghai.

### Western blotting

THP-1 macrophages were infected with EV71 in RPMI 1640 medium without FBS. Cell lysates and chloroform-methanol concentrated supernatants were separated by SDS-PAGE, transferred to nitrocellulose membranes and hybridized with various primary antibodies. Rabbit anti-EV71 VP1 polyclonal antibody was generated previously^37^. Rabbit anti- ASC (sc-22514, Santa Cruz), rabbit anti-human caspase-1 (sc-515, Santa Cruz), rabbit anti- mature and pro-IL-1β (sc-7884, Santa Cruz), mouse anti-β-actin (Sigma), as well as appropriate HRP-conjugated secondary antibodies (Santa Cruz) were applied for signal detection via ECL reagent (Perkin Elmer). ASC oligomerization detection was performed as described before^25^.

### In vivo influenza A virus infection

6–8-week old female C57BL/6 mice from Shanghai Laboratory Animal Center were employed for in vivo experiments. Animals were housed under SPF conditions with 12 h light and dark cycles before transfer to Animal Biosafety Level 2 (ABSL2) laboratory for infection assay. Mice were randomly divided into two groups for PR8 inoculation and PBS/FAC administration. Anesthetized by 2.4% avertin, mice were intranasally challenged with 1 × LD_50_ PR8 diluted in 25 μl PBS or PBS with FAC (15 mM). Mice body weight was recorded each day for 15 days. Mice with relative weight lower than 75% of original weight were euthanatized and considered as death.

For whole lung RNA isolation, mice were sacrificed and the lung was dissected and homogenized in TRIzol. The homogenate was centrifuged at 15,000×*g*, 4 °C for 10 min. The supernatant was then used for RNA extraction.

For lung BALF analysis, mice were anesthetized and thorax opened. 1 ml FACS buffer (PBS + 0.5% BSA) was injected into lung through trachea. The lung was washed three times by FACS buffer to get BALF. After 400×*g*, 4 °C, 5 min centrifugation, supernatant was subjected to ELISA analysis for IL-1β, IL-6, TNF-α, and CCL2 (eBiosciences). Cells were resuspended and washed by FACS buffer and were preincubated with anti–CD16/CD32 mAb to block FcγRII/III receptors and stained with APC-Ly-6G mAb (17-5931-81, eBioscience). Cells were analyzed on flow cytometer (Fortessa, BD Biosciences). Data were analyzed via FlowJo software (TreeStar).

For pathology analysis, whole lung was fixed in 4% paraformaldehyde (PFA), dehydrated, paraffin embedded, sectioned, rehydrated and stained with hematoxylin and eosin (H&E).

Animal care and use and experimental procedures complied with national guidelines and were approved by the animal care and use committee at Institut Pasteur of Shanghai.

### Real-time PCR

The extracted RNA was quantified by spectrophotometer (Nano-100, ALLSHENG) and reverse transcribed to cDNA using GoScript Reverse Transcription System (A5001, Promega) according manufacturer’s instructions. Real-time PCR was performed using SYBR Green Real-time PCR Master Mix (QPK-201, Toyobo) and the 7900HT Real-Time PCR System (Applied Biosystems). For each sample, the normalized amount of target mRNA (N_T_) was calculated from the obtained CT values of both target and GAPDH mRNA with the following equation: N_T_ = 2^CT of GAPDH−CT of target^. The primers used are listed below.

**Table:** 

Primers used for real-time PCR		
hGAPDH	GGTATCGTGGAAGGACTCATGAC	ATGCCAGTGAGCTTCCCGTTCAGC
PR8	AACCGCAAATGCAGACAC	CAGAGTGTGTTACTGTTACATTCTT
HIV-1	GGAGCTAGAACGATTCGCAGTTA	GGTTGTAGCTGTCCCAGTATTTGTC
Zika virus	CAACCACTGCAAGCGGAAGGGT	AAGTGATCCATGTGATCAGTTGA
EV71	TGAATGCGGCTAATCCCAACT	AAGAAACACGGACACCCAAAG
hIFN-β	AGGACAGGATGAACTTTGAC	TGATAGACATTAGCCAGGAG
hIL-1β	CACGATGCACCTGTACGATCA	GTTGCTCCATATCCTGTCCCT
hIL-6	CCTGAACCTTCCAAAGATGGC	TTCACCAGGCAAGTCTCCTCA
mGAPDH	AGGTCGGTGTGAACGGATTTG	TGTAGACCATGTAGTTGAGGTCA
mIL-6	TAGTCCTTCCTACCCCAATTTCC	TTGGTCCTTAGCCACTCCTTC
mTNF-α	GGAACACGTCGTGGGATAATG	GGCAGACTTTGGATGCTTCTT
mCCL2	TTAAAAACCTGGATCGGAACCAA	GCATTAGCTTCAGATTTACGGGT
mIFN-β	AGCTCCAAGAAAGGACGAACA	GCCCTGTAGGTGAGGTTGAT
mIFN-γ	GCCACGGCACAGTCATTGA	TGCTGATGGCCTGATTGTCTT
mKC	CTGGGATTCACCTCAAGAACATC	CAGGGTCAAGGCAAGCCTC

### ELISA

Human IL-1β in supernatants of THP-1 macrophages was quantified via eBiosciences ELISA kit (88–7261). Mouse IL-1β, IL-6, TNF-α, and CCL2 from lung BALFs were analyzed via eBiosciences ELISA kits (88–7013, 88–7064, 88–7324, and 88–7391, respectively). All the procedures were performed according to the manufacturer’s instructions.

### Immunofluorescence and confocal microscopy

A549, Hela or RD cells were seeded on circle glass coverslips and treated as indicated. Cells were fixed in 4% PFA in PBS for 30 min at room temperature (RT) and permeabilized with 0.1% Triton X-100 (Sigma) in PBS for 10 min at room temperature (RT). Cells were blocked in 10% goat serum for 1 h (RT) and stained with mouse anti-nucleoprotein (NP) (MAB8258, Merck Millipore), mouse anti-actin (Sigma), rabbit anti-RAB5 (3547S, Cell Signaling) or rabbit anti-RAB7 (9367S, Cell Signaling) at 4 °C overnight. Cells were then stained with fluorescent secondary antibodies, Alexa Fluor-488 Goat anti-Mouse IgG (A11029), Alexa Fluor-488 Goat anti-Rabbit (A11034) or Alexa Fluor-555 Goat anti-Mouse (A21424) (Thermo Fisher Scientific) for 1 h in dark (RT). Coverslips were then mounted by DAPI containing mountant (P36966, Thermo Fisher Scientific) at 4 °C in dark over night before microscopy. Leica DMI3000 B was used for traditional fluorescence microscopy and Olympus FV-1200 was used for confocal microscopy. Images were captured using manufactures’ software.

For Alexa Fluor-555 conjugated transferrin (T35352, Thermo Fisher Scientific) and Cholera Toxin Subunit B (CTB, C22843, Thermo Fisher Scientific) treatment and imaging, A549 cells were treated for 1 h, fixed, mounted and then subjected to confocal microscopy.

For quantitative analysis of RAB7^+^ vesicle areas, confocal microscopy images captured under the same machine and software settings were analyzed by Image-Pro Premier 9.2 software. RAB7 vesicle average areas and total vesicle areas per cell were calculated according to RAB7 color (red) intensity.

For imaging analysis of RAB7-GFP overexpressing Hela cells, the cells were transfected by pFUGW-RAB7-GFP (a gift from Prof. Ying Wan, Third Military Medical University, Chongqing, China) for 40 h, infected by PR8 for 6 h, and stained with anti-NP and Alexa Fluor-555 Goat anti-Mouse antibodies before confocal microscopy.

All image data shown were representative of at least five randomly selected fields from at least three independent experiments.

### Electron microscopy

A549 cells grown in 6-well plates were infected with PR8 (MOI = 0.5) for 1 h or 6 h with or without FAC (100 μM). Cells were washed with PBS, treated with 0.2 ml trypsin (25300062, Thermo Fisher Scientific) for 1–2 min and stopped. Cells were then transferred to 1.5 ml centrifuge tube, washed by prechilled PBS once, centrifuged to pellet, and fixed by 2.5% glutaraldehyde (in 0.1 M PBS) for 1 h at RT followed by further fixation at 4 °C overnight. After post-fixation by 1% osmium tetroxide (in 0.1 M PBS) for 1 h, cells were dehydrated with graded ethanol solutions (30, 50, 70, 80, 95, and 100%) and sodium sulfate anhydrous treated acetone. Specimens were infiltrated sequentially with a 1:1 mixture of acetone and EPON resin for 1.5 h, and 100% EPON resin overnight. Specimens were then embedded in embedding molds and polymerized for 48 h in an oven at 60 °C. Thin sections (60–90 nm) were cut using a Leica ultramicrotome, mounted on copper grids, and stained with uranyl acetate and lead citrate. Transmission electron microscopy images were captured using FEI Tecnai G2 Spirit Twin.

For negative-stain EM of PR8 virus, 0.5 ml (0.5 × 10^7^ pfu/ml) PR8 viruses in DMEM were treated with or without FAC (100 μM) and incubated for 1 h at RT. After 4 °C, 15,000×*g*, 30 min centrifugation, viruses were resuspended by 10 μl 0.01% BSA in ddH_2_O. 2 μl of viruses were added onto copper grid and fixed by 2.5% glutaraldehyde for 10 min. After 10 μl of 2% phosphotungstic acid (PTA, pH7.3) staining for 1 min, the grid was put into grid box allow air-dry overnight before observation.

All image data shown were representative of at least three randomly selected fields from at least two independent experiments.

### HA-mediated cell fusion

Hela cells were transfected with the pFUGW-HA plasmid using lipofectamine 2000. 48 h later, the cells were treated with TPCK-treated trypsin (2.5 μg/ml) for 10 min at 37 °C to cleave HA into HA1 and HA2. Then, cells were treated with DMEM (pH4.8) with or without FAC for 10 min at 37 °C before changed to normal medium (with or without FAC) for 3 h. Syncytia formation was observed after fixation with 4% PFA and stained with 0.1% crystal violet.

### Bright field microscopy and cell morphology analysis

For bright field microscopy of liposome, lipofectamine 2000 (Invitrogen) was diluted into Opti-MEM (Invitrogen) for 30 min at RT to allow formation of liposome. The mix was then transferred into DMEM in 24-well plates with or without FAC. The samples were incubated for 1 h at 37 °C and stained with DiD (50 μM, D7757, Thermo Fisher Scientific) before bright field microscope (Olympus IX73) observation.

For bright field cell morphology observation, A549 or Hela cells were seeded into 24-well plates and treated with Dynasore and/or FAC for indicated time (50 μM and/or 100 μM for 3 h, 80 μM and/or 100 μM for 6 h or 12 h) in DMEM without FBS. Cells were then fixed by 4% PFA, stained with 0.1% crystal violet and analyzed by Olympus IX73 microscope.

All image data shown were representative of at least five randomly selected fields from at least three independent experiments.

### Statistical analysis

GraphPad Prism 5.0 software was used for statistical analysis. Data shown are mean ± SEM or SD as indicated in figure legends. Statistical significance was determined by two-tailed Student’s *t*-test for two groups or two-way ANOVA or log-rank (Mantel–Cox) test. Survival curves were generated via the product-limit method of Kaplan and Meier. In all cases, *P* values < 0.05 were considered statistically significant. *P < 0.05, **P < 0.01, ***P < 0.001.

## Electronic supplementary material

Supplementary Information
