# Supplementary material for: Antiviral effects of ferric ammonium citrate
Source: Cell Discov. 2018 Mar 27;4:14. doi: 10.1038/s41421-018-0013-6 (PMC5871618; doi:10.1038/s41421-018-0013-6)
Supplement: Supplementary file 1 — Supplementary Information [file 41421_2018_13_MOESM1_ESM.pdf]

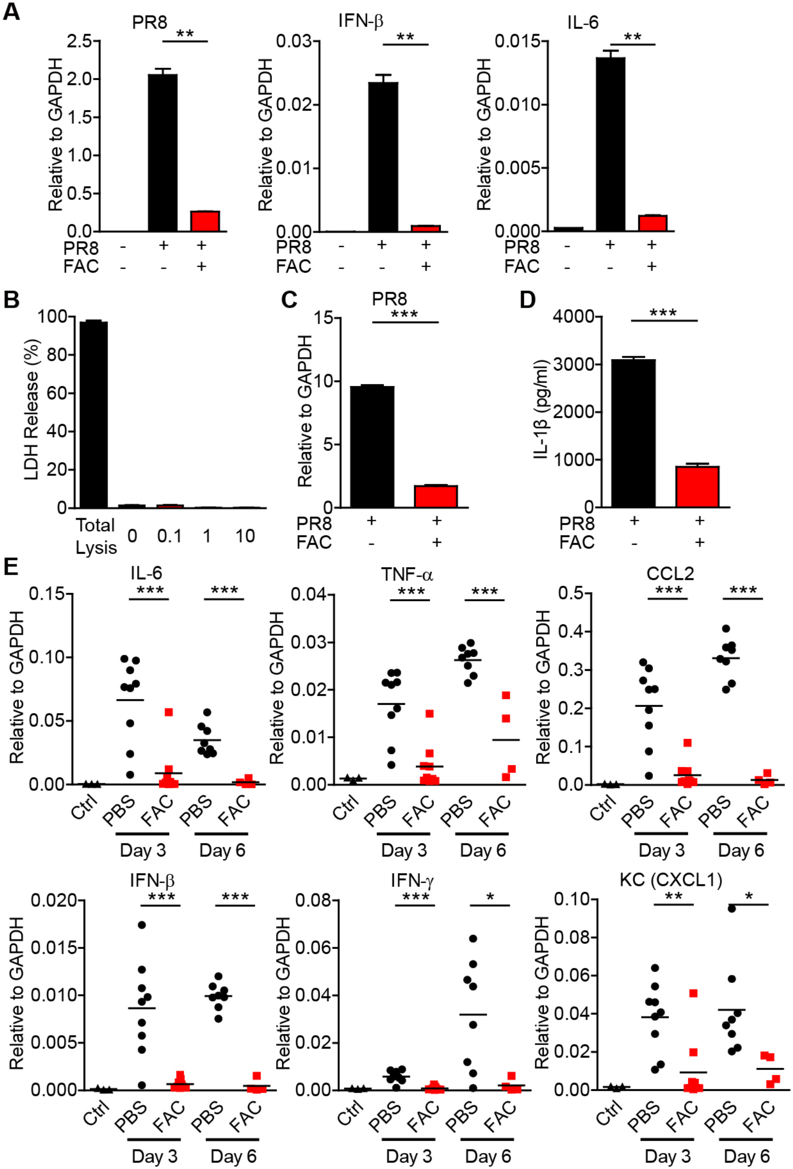

A
PR8
IFN-β
IL-6
Relative to GAPDH
PR8
FAC
B
LDH Release (%)
Total Lysis
0
0.1
1
10
C
PR8
Relative to GAPDH
PR8
FAC
D
IL-1β (pg/ml)
PR8
FAC
E
IL-6
TNF-α
CCL2
IFN-β
IFN-γ
KC (CXCL1)
Relative to GAPDH
Ctrl
PBS
FAC
Day 3
Day 6

Figure S1. FAC inhibited influenza A virus infection.

(**A**) A549 cells were infected with PR8 (MOI=0.1) ± FAC (100 μM). 12 h later, PR8 viral RNA, IFN-β and IL-6 expression were analyzed through real-time PCR. (**B**) A549 cells were treated with increasing concentrations of FAC (0-10 mM). 48 h later, LDH in supernatants were analyzed. (**C** and **D**) THP-1 macrophages were infected with PR8 (MOI=0.1) ± FAC (100 μM). 24 h later, viral RNA was analyzed through real-time PCR (**C**); secreted mature IL-1β in supernatant was analyzed via ELISA (**D**). (**E**) Mice were infected and treated as in **Figure 1E**, whole lung was collected at days 3 and 6 for real-time PCR analysis of IL-6, TNF-α, CCL2, IFN-β, IFN-γ and KC (CXCL1). Error bars represent SD. *P < 0.05, **P < 0.01, ***P <0.001. Two-tailed Student's t-test.

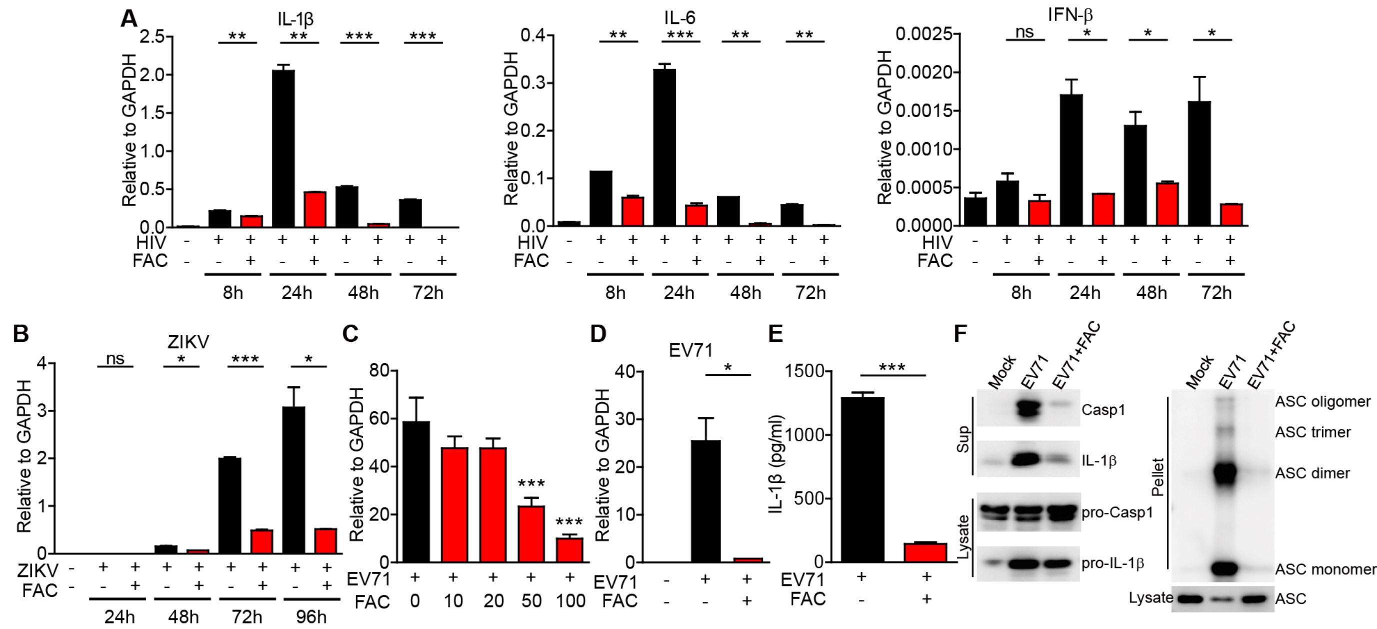
A
IL-1β
IL-6
IFN-β
Relative to GAPDH
HIV
FAC
8h
24h
48h
72h
ns
B
ZIKV
C
D
EV71
E
IL-1β (pg/ml)
FAC 0 10 20 50 100
F
Mock
EV71
EV71+FAC
Sup
Lysate
Casp1
IL-1β
pro-Casp1
pro-IL-1β
Pellet
ASC oligomer
ASC trimer
ASC dimer
ASC monomer
ASC
24h
48h
72h
96h

Figure S2. FAC inhibited viral infection induced innate immune responses.

(**A**) Human PBMC-differentiated dendritic cells were infected with HIV-1 (Gag/p24 100 ng/ml) ± FAC (100 μM). 8, 24, 48 and 72 h later, IL-1β, IL-6 and IFN-β expression was analyzed through real-time PCR. (**B**) Vero cells were infected with Zika virus (MOI=0.1) ± FAC (100 μM). 24, 48, 72 and 96 h later, viral RNA was analyzed through real-time PCR. (**C**) RD cells were infected with EV71 (MOI=0.1), treated with increasing doses of FAC (μM) for 8 h. Viral RNA was detected via real-time PCR. (**D and E**) THP-1 macrophages were infected with EV71 (MOI=0.1) ± FAC (100 μM). 24 h later, viral RNA was analyzed through real-time PCR (**D**); IL-1β in the supernatant was analyzed through ELISA (**E**). (**F**) THP-1 macrophages were infected and treated with FAC as in (**D**). 24 h later, IL-1β and Caspase-1 (Casp1) from supernatant (Sup) and ASC oligomer formation in cell pellets were analyzed via western blotting. Data are representative of two independent experiments. Error bars represent SD. *P < 0.05, **P < 0.01, ***P <0.001. Two-tailed Student's t-test.

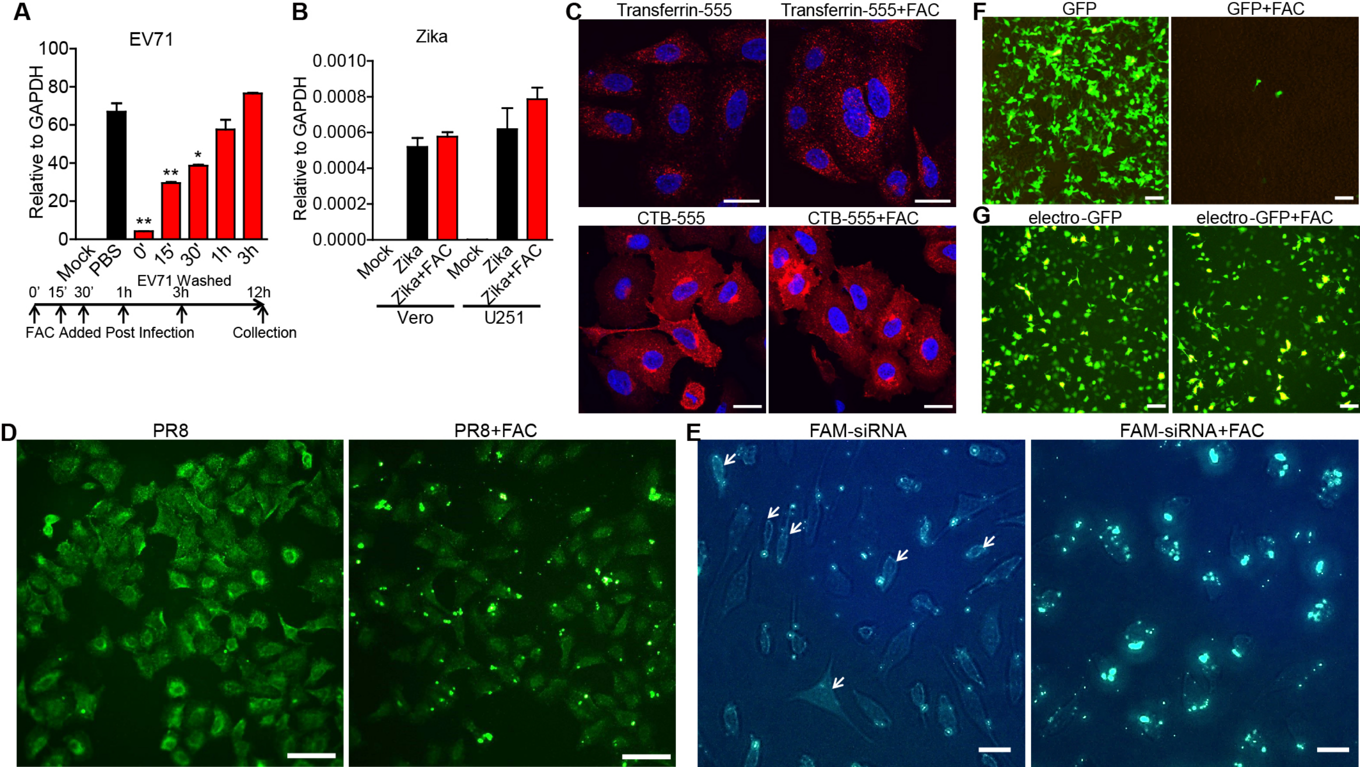

A
EV71
Relative to GAPDH
100
80
60
40
20
0
**
**
*
Mock
PBS
0'
15'
30'
1h
3h
EV71 Washed
0'
15'
30'
1h
3h
12h
FAC Added Post Infection
Collection
B
Zika
Relative to GAPDH
0.0010
0.0008
0.0006
0.0004
0.0002
0.0000
Mock
Zika
Zika+FAC
Mock
Zika
Zika+FAC
Vero
U251
C
Transferrin-555
Transferrin-555+FAC
CTB-555
CTB-555+FAC
F
GFP
GFP+FAC
G
electro-GFP
electro-GFP+FAC
D
PR8
PR8+FAC
E
FAM-siRNA
FAM-siRNA+FAC

Figure S3. FAC inhibited early viral infection and induced fusion of influenza A virus or liposome.

(**A**) EV71 infected RD cells were treated with FAC at indicated time points. Viruses were washed out at 3 h and cells were analyzed for viral RNA at 12 h post infection through real-time PCR. (**B**) Vero and U251 cells were infected with Zika virus ± FAC for 1 h at 0℃. Cells were washed and analyzed for viral RNA through real-time PCR. (**C**) A549 cells were treated with Alexa Fluor-555 conjugated transferrin (10 μg/ml) or Cholera Toxin Subunit B (CTB, 5 μg/ml) ± FAC (100 μM) for 1 h. Cells were analyzed under confocal microscopy. Scale bars, 20 μm. (**D**) A549 cells were infected with PR8 ± FAC for 1 h. Cells were analyzed via fluorescence microscopy for PR8 NP protein. Scale bars, 100 μm. (**E**) L929 cells were incubated with liposome and carboxyl fluorescein (FAM)-labeled siRNA (FAM-siRNA) mix ± FAC (50 μM) for 6 h. FAM-siRNA was analyzed by fluorescence microscopy. Arrows indicate FAM-siRNA dispersed in cells. FAC treatment induced larger FAM-siRNA-liposome aggregations without FAM-siRNA dispersion. Scale bars, 50 μm. (**F**) 293T cells were incubated with liposome-GFP plasmid mix ± FAC (50 μM) for 6 h. 48 h later, GFP expression was analyzed by fluorescence microscopy. Scale bars, 50 μm. (**G**) L929 cells were electroporated with GFP plasmid, then treated with or without FAC (50 μM). 48 h later, GFP expression was analyzed by fluorescence microscopy. Scale bars, 50 μm. Data are representative of three independent experiments. Error bars represent SD. *P < 0.05, **P < 0.01, ***P <0.001. Two-tailed Student's t-test.

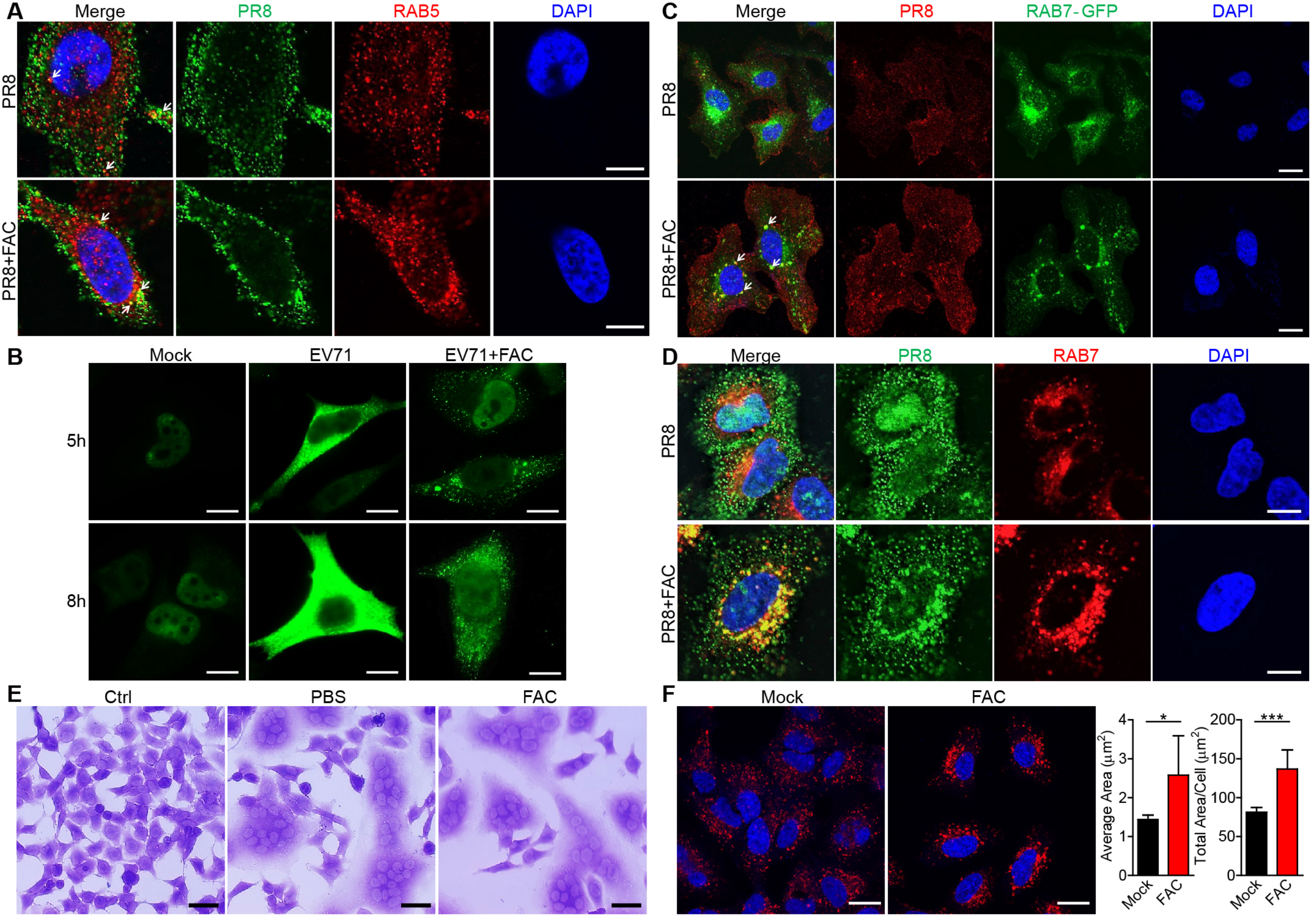

A
Merge
PR8
RAB5
DAPI
PR8
PR8+FAC
B
Mock
EV71
EV71+FAC
5h
8h
C
Merge
PR8
RAB7-GFP
DAPI
PR8
PR8+FAC
D
Merge
PR8
RAB7
DAPI
PR8
PR8+FAC
E
Ctrl
PBS
FAC
F
Mock
FAC
Average Area (μm²)
0
1
2
3
4
*
Mock
FAC
Total Area/Cell (μm²)
0
50
100
150
200
***
Mock
FAC

Figure S4. FAC induced intracellular vesicle fusion and inhibited viral endosomal release.

(**A**) Hela cells were infected with PR8 at 4℃ for 20 min before washing out viruses, then PBS or FAC were added for 15 min at 37℃. Cells were analyzed by confocal microscopy for PR8 NP protein and RAB5. Arrows indicate co-localizations. Scale bars, 10 μm. (**B**) EV71 infected Hela cells were treated with or without FAC for 5 h or 8 h. Cells were stained with anti-EV71 polyclonal antibody and secondary fluorescent antibody, then visualized under fluorescence microscopy. Scale bars, 10 μm. (**C**) RAB7-GFP plasmid transfected Hela cells were infected with PR8 ± FAC for 6 h. Cells were analyzed by confocal microscopy for PR8 NP protein and RAB7-GFP. Arrows indicate co-localizations. Scale bars, 20 μm. (**D**) PR8 infected A549 cells were treated with or without FAC for 6 h. Cells were analyzed by confocal microscopy for PR8 NP protein and RAB7. Scale bars, 10 μm. (**E**) Hela cells with (PBS, FAC) or without (Ctrl) PR8 HA protein overexpression were subjected to HA-mediated cell fusion treatment in the presence or absence of FAC. Cells were stained with 0.1% crystal violet and analyzed via bright field microscopy. Scale bars, 50 μm. (**F**) A549 cells were treated with or without FAC for 6 h and were analyzed through confocal microscopy for RAB7. Average RAB7 positive vesicle area and total RAB7 positive vesicle area per cell were statistically analyzed. Scale bars, 20 μm. Data are representative of three independent experiments. Error bars represent SD. $^*P < 0.05$, $^{**}P < 0.01$, $^{***}P < 0.001$. Two-tailed Student's t-test.

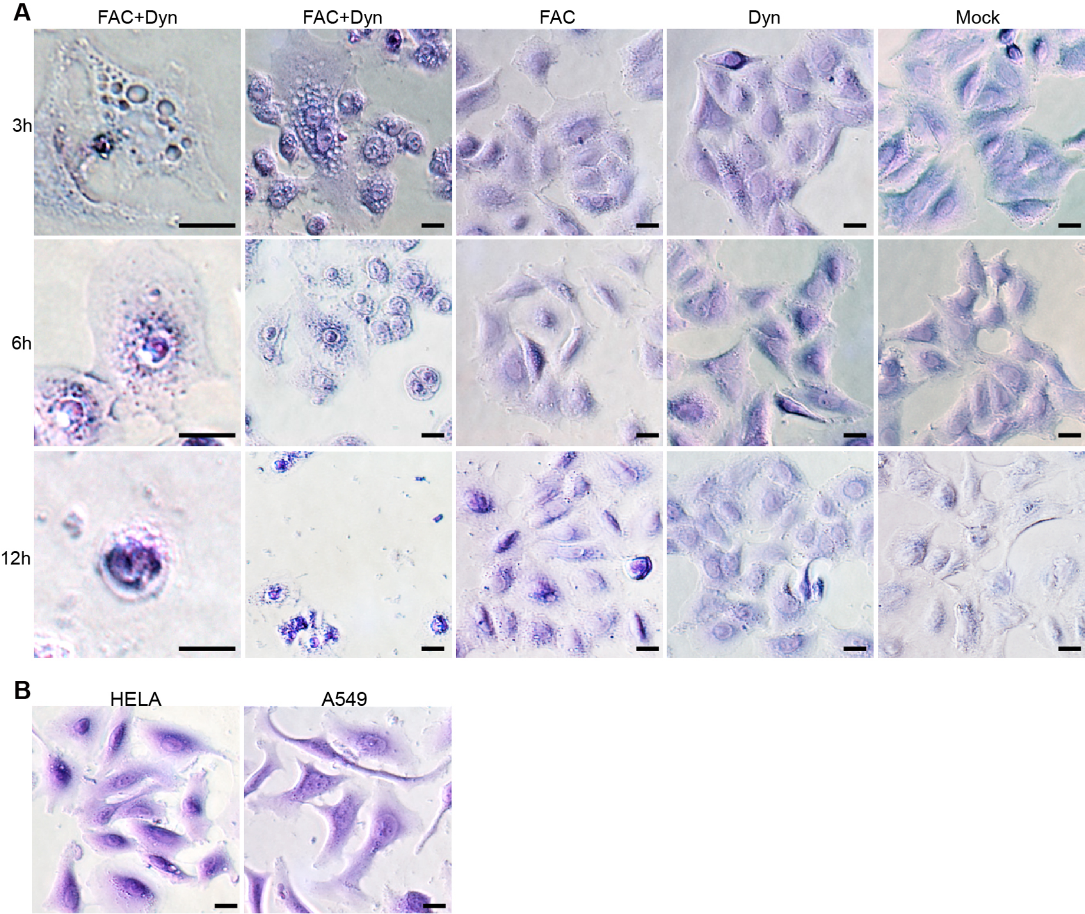
A
FAC+Dyn
FAC+Dyn
FAC
Dyn
Mock
3h
6h
12h
B
HELA
A549

Figure S5. FAC combining with Dynasore induced an iron dependent cell death.

(**A**) A549 cells were treated with Dyn (50 μM 3 h, 80 μM 6 h or 12 h) and/or FAC (100 μM) for indicated time. Cells were stained with 0.1% crystal violet and analyzed via bright field microscopy. Scale bars, 20 μm. (**B**) Hela and A549 cells were treated with Dyn and FAC for 6 h before washing and changing to normal culture with regular media. 24 h later, cells were crystal violet stained and analyzed via bright field microscopy. Scale bars, 20 μm. Data are representative of three independent experiments. Error bars represent SD. *P < 0.05, **P < 0.01, ***P <0.001. Two-tailed Student's t-test.
